# Sugar-sweetened beverage affordability and the prevalence of overweight and obesity in a cross section of countries

**DOI:** 10.1186/s12992-019-0474-x

**Published:** 2019-04-18

**Authors:** Fabrizio Ferretti, Michele Mariani

**Affiliations:** School of Social Sciences, Department of Communication and Economics, University of Modena and Reggio Emilia, Palazzo Dossetti, Viale Allegri, 9, 42121 Reggio Emilia, Italy

**Keywords:** Affordability, Globalization, Obesity, Overweight, Soft drinks, Sugar-sweetened beverages

## Abstract

**Background:**

A key component of ‘obesogenic environments’ is the ready availability of convenient, calorie-dense foods, in the form of hyper-palatable and relatively inexpensive ultra-processed products. Compelling evidence indicates that the regular consumption of soft drinks, specifically carbonated and non-carbonated sugar-sweetened beverages (SSBs), has a significant impact on the prevalence of overweight and obesity. However, to implement country-level effective prevention programmes we need to supplement this evidence with quantitative knowledge of the relationships between overweight/obesity and the main determinants of SSB consumption, notably SSB prices and consumers’ disposable income.

**Method:**

Affordability considers the simultaneous effects of both price and disposable income on the buying decision. The purpose of this study was to investigate the effect of SSB affordability on the consumers’ purchasing behaviour and weight-related health outcomes. Our study was divided into three parts. First, we computed SSB consumption and affordability for approximately 150 countries worldwide. Second, we estimated a demand function for SSBs to assess the impact of affordability on consumption at the country level. Third, we used a multivariate regression model and country data on the prevalence of overweight and obesity to test the role of SSB affordability in the current obesity epidemic.

**Results:**

The analysis reveals that SSB affordability: 1) showed both a large variability across countries and a clear tendency to increase substantially with the level of economic development; 2) played a key role in determining cross-country differences in the amount of soft drink consumed per capita; and 3) was significantly associated with the prevalence rates of both overweight and obesity. Specifically, we show that a 10 % increase in SSB affordability was associated, on average, with approximately 0.4 more overweight/obese adults per 100 inhabitants.

**Conclusions:**

By controlling for the main possible confounding factors, our results clearly indicate that affordability is a major driver of purchasing behaviours and is significantly associated with the prevalence rates of both overweight and obesity. We thus suggest a fiscal approach to curb SSB consumption based on the effectiveness of ‘soda taxes’ to affect the long-term dynamic of SSB affordability.

**Electronic supplementary material:**

The online version of this article (10.1186/s12992-019-0474-x) contains supplementary material, which is available to authorized users.

## Background

Overweight and obesity are usually defined as an abnormal or excessive body fat accumulation that might seriously impair people’s health [1]. Overweight and obesity are indeed major risk factors for many severe non-communicable diseases, such as cardiovascular diseases, diabetes, musculoskeletal disorders, and also some types of cancers [2].

It is widely acknowledged that, although they are complex and multifactorial conditions, overweight and obesity would be largely preventable through ‘relatively simple’ lifestyle changes [3, 4]. Despite this, the worldwide prevalence of overweight and obesity has increased substantially during the last decades [5]. Nowadays, it is estimated that approximately two-thirds of the world’s population lives in countries where there are more deaths attributable to overweight and obesity-related diseases than to underweight and malnutrition [6]. If recent trends continue unchanged, the latest projections suggest that by 2030 up to 57.8% of the world’s adult population could be either overweight or obese [7].

An abnormal or excessive body fat accumulation results from a sustained positive energy imbalance between calories consumed and calories expended [8]. Besides the role of heredity, this energy imbalance comes from the adoption of lifestyles characterized by an unhealthy diet and/or a lack of physical activity [9]. Research has shown that the spread of overweight and obesity in high- and middle-income countries is mainly driven by structural changes that affect these health-related habits and behaviours [10].

In particular, in many countries the intensive use of sophisticated food processing technologies, aggressive food marketing strategies, and the pervasive diffusion of ICT technologies and automation (along with urbanization, aging, and other cultural, economic, and social transformations), have developed into an ‘obesogenic environment’ [11, 12], that is a society that tends to promote unhealthy weight gain by pushing people towards overweight and obesity-prone lifestyles [13].

A key component of these ‘obesogenic environments’ is the high availability of convenient (i.e. durable and ready to consume), calorie-dense foods in the form of hyper-palatable and relatively inexpensive, ultra-processed products [14, 15]. These foods are typically low in nutrients and high in added fat and sugars, and their daily consumption is strongly associated with a higher risk of becoming overweight or obese [16, 17].

In addition to ultra-processed foods, an important source of ‘empty calories’ are soft drinks—specifically carbonated and non-carbonated sugar-sweetened beverages (SSBs), such as sodas and ready-to-drink tea, fruit and fruit flavored beverages—that usually contain large amounts of refined sugars (most often high-fructose corn syrup) but few or no nutrients^1^ [18, 19].

The worldwide demand for these beverages has grown rapidly over the last decades [20, 21]. Despite some recent signs of a trend reversal in North American and European countries (especially for non-diet carbonated soft drinks), the per capita consumption of SSBs still remains high in both middle- and high-income countries and is predicted to rise in the near future, especially in populous and fast-growing economies such as China and India [22].

Compelling evidence from observational studies and experimental trials indicates that the regular consumption of SSBs has a significant impact on the prevalence of overweight and obesity (in both children and adults) and contributes substantially to the onset of other metabolic diseases (notably, type 2 diabetes) [23–25]. To implement country-level effective overweight and obesity prevention programmes, however, this evidence should be supplemented with thorough quantitative knowledge of the relationships between overweight/obesity and the main determinants of SSB consumption, notably SSB prices and consumer disposable income [26, 27].

So far a lack of reliable and comparable country data on both overweight and obesity prevalence and SSB sales and prices has limited the application of ecological studies to this public health issue. More recently, however, by taking advantage of new cross-country data sets, some studies have found that carbonated soft drink consumption is significantly linked to overweight and obesity worldwide [28], whereas others have restricted this link to low- and middle-income countries and have also found little or no robust evidence of the effects of soft drink prices on unhealthy weight gain indicators [29].

These mixed results suggest the need for further research. In the pages that follow, we build on such previous studies to fill a specific research gap in the existing literature. Among the open questions, there is a lack of knowledge about the role of SSB affordability on consumers’ purchasing behaviour and weight-related health outcomes [30].

Broadly speaking, affordability considers the simultaneous effects of both price and disposable income on a consumer’s buying decision and thus captures the consumer’s ‘ability to purchase a specific good or service [31]. The concept of affordability is commonly used to investigate the demand for other important health-related goods, such as alcohol and tobacco [32, 33].

In a nutshell, the rest of this paper represents an attempt to answer a single and high-impact research question: does SSB affordability affect overweight and obesity prevalence worldwide? To answer this, we proceeded in three steps. First, we used the latest statistics available on the beverage market to compute the per capita consumption and comparable measures of SSB prices and affordability for a large number of countries (approximately 150 countries, from all world regions, and with different levels of economic development). Next, we estimated a demand function for SSBs to investigate the impact of affordability on consumption at the country level. Finally, we compared country data on SSB consumption and affordability with data on the prevalence of overweight and obesity to test whether the affordability of SSBs should be included among the key drivers of the so-called ‘obesity pandemic’.

### Data

The data utilised in this study were obtained from six main sources: Euromonitor International, the World Health Organization (WHO), the United Nations’ Food and Agriculture Organization (FAO) and Human Development Report (HDR), the World Bank, and the Swiss Economic Institute (Konjunkturforschungsstelle, KOF).

Euromonitor International regularly updates a comprehensive database on the beverage industry [34]. This database contains information on soft drink sales, in both volume and value, for a large number of countries worldwide. Using these data, we first computed the per capita annual consumption of SSBs (*Q*) in each country by dividing the total sales in volume of non-carbonated (i.e. ready-to-drink tea, coffee, and juices, as well as sports/energy and Asian drinks^2^) and carbonated soft drinks by the total country population. *Q* includes on-trade and off-trade sales of both domestically manufactured and imported beverages. Population data were taken from the United Nations HDR database [35].

Second, we obtained an average market price of SSBs (*P*) at the country level by dividing total sales in value by total sales in volume. These average annual prices, expressed in local currency, were converted to a common currency using purchasing power parity (PPP) conversion factors from the World Bank’s International Comparison Program database [36]. In a similar way, using Euromonitor data, we computed the average annual price and per capita consumption of bottled (still and carbonated) water, respectively denoted as *PW* and *QW* [34].

Within the Global Health Observatory, the WHO provides comparable estimates of the prevalence of overweight and obesity for almost all countries worldwide [37]. From this data repository, we retrieved the age-standardized adjusted estimates of the prevalence of overweight (*POW*) and obesity (*POB*) among the adult population. *POW* and *POB* are measured by the percentage of adults (aged 18+ years) who have a body mass index (BMI) equal to or greater than 25 kg/m^2^ or 30 kg/m^2^, respectively (where BMI is defined as weight in kilograms divided by the square of height in meters).

To isolate the impact of SSB consumption and affordability on population-wide unhealthy weight gains, we included in our database a number of control variables that previous research has linked to the spread of overweight and obesity. Increased food energy supply has been proven to be a key driver of the obesity epidemic [38]. Dietary energy supply (*DES*, expressed in kcal/person/day) is a common measure of the average amount of the food available for human consumption at the country level. We used internationally comparable *DES* estimates, as computed by the FAO from national balance food sheets [39].

Urbanization and economic structural changes affect people’s health-related habits and behaviours, influencing the prevalence of overweight and obesity [40]. We tried to capture the impact of these factors by including the percentage of the total population that lives in urban areas (*URB*), as well as the percentage of total employment allocated to services (*ESE*), (both *URB* and *ESE* were taken from the HDR database [35]).

The United Nations HDR [35] also provides data on the following three variables: 1) the number of physicians per 10,000 people (*PHY*); 2) a widely accepted country measure of gender inequality, the Gender Inequality Index (*GII*); and 3) the gross national income per capita (*YPC*), expressed in international dollars (PPP exchange rates) for comparability.

We used *PHY* and *GII* as proxy variables to account for country differences in the quality of health statistics registration and reporting [41], and the health, empowerment, and economic status of the female population, respectively. Indeed recent evidence suggests a significant direct relationship between the prevalence of overweight and obesity among women and the level of gender discrimination [42, 43]. Income per capita (*YPC*) is included in our dataset as both a determinant of soft drink consumption and a key variable to compute soft drink affordability.

Finally, in the soft drink industry a few large international corporations control a highly globalized market [21], promoting changes in traditional dietary patterns and a convergence towards ‘Western-style’ eating habits [44–46]. These cultural and social transformations have been found in previous research to contribute to the rising obesity problem in lower- and middle-income countries [47]. We thus computed a basic country indicator of a ‘Westernized lifestyle’ to adjust our analysis for these potential confounding factors. This indicator, denoted *WLS*, is based upon the geometric mean of the KOF [48] Globalization Index (*GLO*)—a summary measure of a country’s degree of economic, political, and social globalization—and the level of urbanization (*URB*).

Overall, all data refer to the years 2014 or 2015. A short description of each variable, along with basic descriptive statistics, is shown in Table 1 (for a full description and the complete database see Tables S1 and S2 in Additional file 1). The correlation coefficients, along with their statistical significance, are shown in Table 2.

**Table 1:** Descriptive statistics

Variable	Description	Mean	Std. dev.	Min	Max	*n*
POW	Prevalence of overweight age-stand. Rate, both sex (BMI ≥ 25), person 18+	47.52	16.75	15.50	78.10	181
POB	Prevalence of obesity age-stand. Rate, both sex (BMI ≥ 30), person 18+	18.79	9.88	2.60	43.40	181
Q	Per capita consumption of sugar-sweetened beverages (Litres/person/year)	56.62	43.69	0.69	236.58	183
QW	Per capita consumption of (bottled still and carbonated) water (Litres/person/year)	48.96	48.00	0.16	205.43	183
YPC	Gross national income per capita, PPP $	17,005.28	17,365.99	587.47	78,162.32	182
P	(Average) Price of sugar-sweetened beverages (PPP $ per litre)	3.17	1.08	0.78	6.79	154
SBA	Sugar-sweetened beverage affordability (SSB relative income price), (per 100 l, %)	6.21	8.76	0.36	42.54	154
PW	(Average) Price of water (bottled still and carbonated), (PPP $ per litre)	1.70	0.88	0.56	8.05	151
DES	Dietary energy supply (Kcal/person/day)	2881.96	444.89	1937.00	3810.00	158
PHY	Number of physicians (per 10,000 people)	16.06	14.83	0.14	67.23	177
GLO	KOF Globalization index	58.48	16.44	23.98	92.84	178
URB	Urban population (% of total population living in urban areas)	56.57	23.22	8.45	100.00	182
GII	UNDP Gender inequality index	0.36	0.19	0.04	0.77	157
ESE	Employment in services (% of total employment)	54.21	17.61	16.20	85.70	182
WLS	Index of ‘Western lifestyle’ (GLO and URB)	0.55	0.20	0.10	0.99	182

**Table 2:** Correlation coefficients

Variable	POW	POB	Q	QW	YPC	P	SBA	PW	DES	PHY	GII	ESE	WLS
POW	1.000												
POB	.957**	1.000											
Q	.641**	.572**	1.000										
QW	.542**	.526**	.559**	1.000									
YPC	.496**	.435**	.716**	.452**	1.000								
P	−.297**	−.289**	−.344**	−.144	−.095	1.000							
SBA	−.676**	−.593**	−.604**	−.458**	−.534**	.285**	1.000						
PW	−.100	−.116	−.034	−.193	.026	.532**	.140	1.000					
DES	.659**	.602**	.620**	.515**	.676**	−.311**	−.623**	−.192*	1.000				
PHY	.567**	.449**	.590**	.429**	.610**	−.125	−.532**	−.043	.719**	1.000			
GII	−.538**	−.416**	−.667**	−.419**	−.751**	.169*	.630**	.003	−.716**	−.787**	1.000		
ESE	.739**	.676**	.747**	.490**	.720**	−.250**	−.739**	.002	.718**	.658**	−.704**	1.000	
WLS	.567**	.436**	.697**	.450**	.669**	−.164*	−.569**	−.004	.709**	.714**	−.728**	.706**	1.000

Note: * and ** denote statistically significant correlation at the 0.05 and 0.01 probability levels (2-tailed), respectively

## Methods

Affordability refers to the quantity of resources, usually measured in terms of time or income, that a consumer needs to sacrifice to acquire a given amount of a specific good or service [31]. Following the current literature on the economics of alcohol [32, 49] and tobacco control [33], in this paper we measured the affordability of SSBs (*SBA*) by the SSB ‘relative income price’. This ratio indicates the percentage of the consumer’s income—measured here by the gross national income per capita—required to buy 100 l of SSBs. In terms of our notation, *SBA* = (100 *PSD*)/*YPC*. As a result, the higher the country’s relative income price, the less affordable SSBs are in that country, and vice versa.

To explore the relationship between SSB affordability and the prevalence of overweight and obesity, the following multivariate regression models were developed. First, we investigated the impact of affordability (*SBA*) on consumption (*Q*). To this aim, we estimated two equations: 1) a standard demand function, including the average price of SSBs (*P*) and the gross national income per capita (*YPC*) separately, along with the price of bottled water (as a related good, i.e. a substitute or a complement) and the ‘Western lifestyle’ index (*WLS*):

1 $$ {Q}_i=\kern0.5em {\beta}_0+{\beta}_1{P}_i+{\beta}_2\ln {YPC}_i+{\beta}_3{PW}_i+{\beta}_4{WLS}_i+{\upvarepsilon}_i $$

and 2) a reformulation of the demand function, in which price and income are combined into the SSB ‘relative income price’ (*SBA*), but *PW* and *WLS* maintain their role of demand shifters:

2 $$ {Q}_i\kern0.5em =\kern0.5em {\beta}_0+{\beta}_1\ln {SBA}_i+{\beta}_2{PW}_i+{\beta}_3{WLS}_i+{\upvarepsilon}_i $$

(here and in the following equations, ln stands for natural logarithm and the subscript *i* denotes the *i*th country).

Second, we isolated the impact of soft drink consumption on the population weight outcomes by regressing the age-standardized prevalence rate of overweight (*POW*) and that of obesity (*POB*) on the quantity of SSBs consumed per capita (*Q*), after adjusting for the following main confounding factors: the amount of food available for human consumption (i.e. the dietary energy supply, *DES*), the percentage of employment in the service sector (*ESE*), the level of gender inequality (*GII*), and the number of physicians per 10,000 people (*PHY*):

3 $$ {POW}_i\kern0.5em =\kern0.5em {\beta}_0+{\beta}_1\ln {Q}_i+{\beta}_2\ln {QW}_i+{\beta}_3\ln {DES}_i+{\beta}_4\ln {ESE}_i+{\beta}_5\ln {GII}_i+{\beta}_6\ln {PHY}_i+{\upvarepsilon}_i $$

Equation 3) is thus estimated twice, once with the prevalence of overweight (*POW*) and once with that of obesity (*POB*) as dependent variables (we denoted these eqs. 3*a* and 3*b*, respectively). Finally, given that the consumption of bottled water (*QW*) should be unrelated to overweight and obesity, *QW* is used in eqs. 3*a* and 3*b* to check whether potentially relevant variables were omitted from the regression model [28, 29].

Third, the determinants of SSB consumption and those of the prevalence of overweight and obesity, taken from eqs. 2) and 3), were included as explanatory variables in a single model to measure the effect of SSB affordability on the prevalence rates of overweight and obesity, by holding all other confounding factors constant. In the case of overweight, we estimated the following regression equation:

4 $$ {POW}_i\kern0.5em =\kern0.5em {\beta}_0+{\beta}_1\ln {SBA}_i+{\beta}_2\ln {PW}_i+{\beta}_3\ln {DES}_i+{\beta}_4\ln {ESE}_i+{\beta}_5\ln {GII}_i+{\beta}_6\ln {PHY}_i+{\upvarepsilon}_i $$

Compared to eq. 2), this equation does not include the index of ‘Western lifestyle’ (*WLS*) to avoid problems of multicollinearity between regressors. Indeed, as shown in the last row of Table 2, the correlation coefficients between *WLS* and the control variables *DES*, *ESE*, *GII*, and *PHY* are, on average, around 0.70 (*p* < 0.01). Again, by changing the dependent variable, we ran two versions of eq. 4), one for the prevalence of overweight (*POW*) and the other for that of obesity (*POB*), denoted respectively 4*a* and 4*b*.

Finally, in all regression models, a semi-log specification was adopted where the relationship between independent and dependent variables was hypothesized to have an ‘increasing at a decreasing rate’ form, and White’s corrected standard errors were chosen to adjust for heteroskedasticity [50].

## Results

In Table 3 countries are clustered by their gross national income per capita, according to the World Bank income groups [51], to provide a first glimpse of SSB consumption, prices, and affordability worldwide. Overall, SSB consumption increased with the level of economic development, but it also showed great variability. In 2015, the average quantity consumed was approximately 61 and 100 l per capita in high- and upper-middle-income countries, respectively. The consumption in high-income countries, however, was approximately four and ten times higher than that observed in lower-middle and low-income countries (around 27 and 9 l per capita, respectively). Data on the quantity consumed showed a very large dispersion even within groups, especially in lower-middle and low-income countries, where the coefficient of variation of consumption per capita was approximately 93% and 75%, respectively.

**Table 3:** Average SSB consumption, price and affordability, and the prevalence of overweight and obesity, by country income level (2015)

		Q		P		SBA		POW		POB	
		Consumption		Price		Affordability		Prevalence overweight		Prevalence obesity	
		Litres/person/year		PPP $ per litre		% of GNI pc per 100 l		Age std. rate, age 18+		Age std. rate, age 18+	
World Bank income group	n	Mean	Std. dev.	Mean	Std. dev.	Mean	Std. dev.	Mean	Std. dev.	Mean	Std. dev.
High-income, HI	57	99.74	35.64	2.86	0.85	0.88	0.42	59.61	9.15	25.14	7.25
Upper-middle income, UMI	53	60.73	26.74	3.23	1.20	2.77	1.32	54.46	10.74	22.79	7.12
Lower-middle income, LMI	45	26.73	24.98	3.35	1.27	7.77	4.29	39.02	15.30	13.96	9.03
Low-income, LI	28	9.09	6.91	3.47	0.81	24.58	9.59	24.28	5.06	6.50	2.42

Note: World Bank country classifications by income level (GNI per capita in US $, Atlas method): GNIpc < 995 = LI; 996 ≤ GNIpc ≤ 3895 = LMI; 3896 ≤ GNIpc ≤ 12,055 = UMI; GNIpc > 12,055 = HI

Conversely, the average price of SSBs—corrected for purchasing power differences across countries—tended to decrease slightly with income per capita and showed less variability within groups than the quantity consumed. As can be seen in the second column of Table 3, the average price of SSBs in 2015 was approximately $3.5 and $3.3 per litre in low- and lower-middle-income countries, respectively. The price decreased to $3.2 in upper-middle-income countries and dropped somewhat to $2.9 per litre in high-income countries.

Given the large disparities in per capita income around the world, these figures about prices imply substantial cross-country differences in SSB affordability. Overall, affordability nearly tripled moving from low- to high-income countries. Specifically, in 2015, the fraction of gross national income per capita required to buy 100 l of SSBs was on average less than 1 % (approximately 0.9%) in high-income countries. This percentage increased to approximately 2.8% in upper-middle-income countries. SSBs became less affordable especially in developing countries, where the fraction of income required to buy 100 l rose sharply until reaching 7.7% and 24.6% in lower-middle and low-income countries, respectively. Here again, a remarkable variability between countries of the same income group was observed.

Table 4 contains a list of the top five countries for SSB consumption in 2015 for each of the six WHO geographic regions [52], along with the corresponding SSB affordability. It is notable that in the Americas, Europe, the Western Pacific regions, and in countries where SSBs were extremely affordable (i.e. the relative income price was, on average, approximately 0.5%), the annual consumption per capita was often well above 100 l (for instance, 167, 150, and 144 l in Mexico, Germany, and Japan, respectively). This implies an average daily consumption of around 0.3–0.4 l per inhabitant.

**Table 4:** SSB consumption and affordability: top five countries by geographic region (2015)

		Q	SBA
		Consumption	Affordability
WHO Region		(Litres/person/year)	(% of GNI pc/100 l)
Americas	United States	236.58	0.36
	Mexico	166.98	0.38
	Chile	161.59	0.44
	Argentina	157.40	0.47
	Canada	154.54	0.48
Europe	Belgium	155.73	0.70
	Germany	150.65	0.49
	Switzerland	143.41	0.54
	Norway	134.79	0.55
	Netherlands	129.16	0.55
Western Pacific	Japan	144.27	0.53
	Australia	131.07	0.55
	Hong Kong	86.07	0.57
	New Zealand	81.60	0.64
	Singapore	76.32	0.49
South-East Asia	Thailand	59.81	2.34
	Maldives	37.86	2.42
	Indonesia	20.23	1.07
	Sri Lanka	10.74	3.07
	Myanmar	5.21	11.47
Eastern Mediterranean	Saudi Arabia	127.53	0.62
	Oman	104.58	1.07
	Kuwait	98.00	0.54
	United Arab Emirates	97.88	0.67
	Bahrain	95.43	1.00
Africa	South Africa	103.24	2.15
	Botswana	70.31	2.05
	Mauritius	47.44	1.64
	Namibia	46.91	3.69
	Algeria	46.09	0.57

**Fig. 1:**
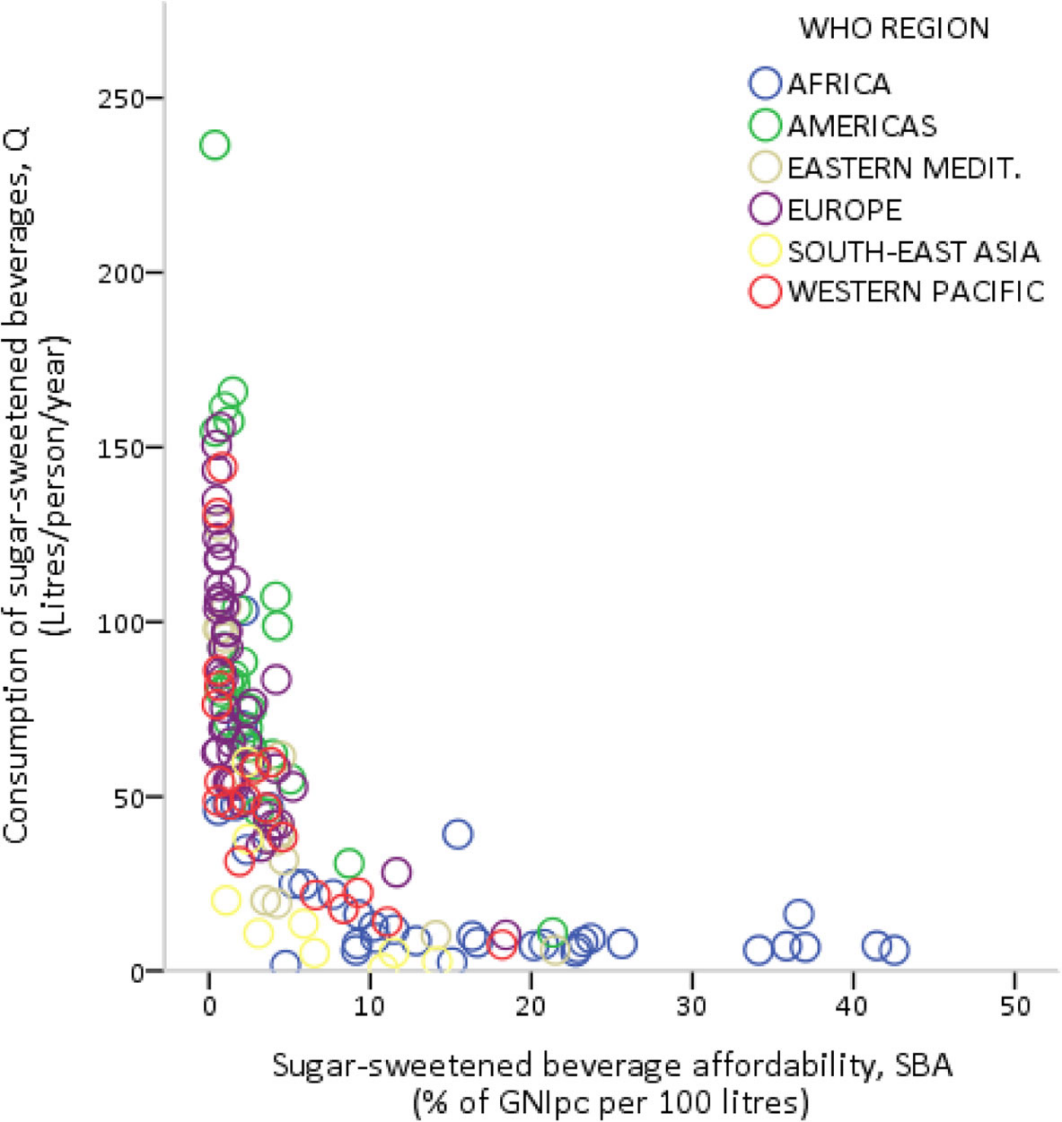
Sugar-sweetened beverage affordability and consumption

The nature of the association between SSB affordability (*SBA*) and consumption (*Q*) is better illustrated in Fig. 1, where *SBA* and *Q* are measured on the x and y axes, respectively. On the one hand, there was a clear inverse relationship between affordability and consumption (*r* = − 0.62; *p* < 0.01, see Table 2). That is, increases in the ‘relative income price’ (i.e. a rightward movement along the x-axis) lead to a decrease in the quantity consumed. On the other hand, despite greater affordability generally increasing the consumption of SSBs, around the same level of affordability (between 0.5% and 1.5%) was associated with very different levels of consumption per capita. This was particularly the case for various countries in Europe and the Americas.

The impact of affordability on consumption was assessed in Table 5, which collects the regression results of eqs. 1) and 2). Despite its simplicity, this demand model was able to explain more than two-thirds of the variation in SSB consumption (the adjusted *R*^2^ is approximately 0.7 in both specifications). Except for the price of bottled water, all estimated coefficients were statistically significant (*p* < 0.01) and had the predicted sign. Specifically, consumption increased with per capita income and ‘Western lifestyle’, whereas price and affordability were inversely related to quantity consumed.

**Table 5:** Regression results: sugar-sweetened beverage demand function

Dependent variable: sugar-sweetened beverage consumption per capita, Q				
Equation (1)		Coefficient	Std. Error^a^	t-statistic
(Average) Price of sugar-sweetened beverages	P	−12.49*	1.98	−6.30
Gross national income per capita	ln(YPC)	21.93*	2.27	9.68
(Average) Price of water	PW	3.32	2.89	1.15
Index of ‘Western lifestyle’	WLS	46.96*	13.48	3.48
Constant = − 135.23, F-statistic = 86.67 (p < 0.01), Adj. R-squared = 0.71, *n* = 150				
Equation (2)		Coefficient	Std. Error^a^	t-statistic
Sugar-sweetened beverage affordability	ln(SBA)	−24.11*	2.18	−11.06
(Average) Price of water	PW	−0.69	2.97	−0.23
Index of ‘Western lifestyle’	WLS	42.06*	12.52	3.36
Constant = 62.91, F-statistic = 109.08 (*p* < 0.01), Adj. R-squared = 0.69, *n* = 150				

Notes: ln(·) is natural log. * Denote *p* < 0.01

^a^White’s heteroskedasticity-adjusted standard errors

What is most notable in these results, as shown by eq. 2), is that the consumption of SSBs, on average, fell by approximately 0.24 l per capita for every 1 % increase in the relative income price. Put differently, if affordability decreases by 10 %, the quantity consumed will drop by approximately 2.4 l per capita, and vice versa.^3^

**Fig. 2:**
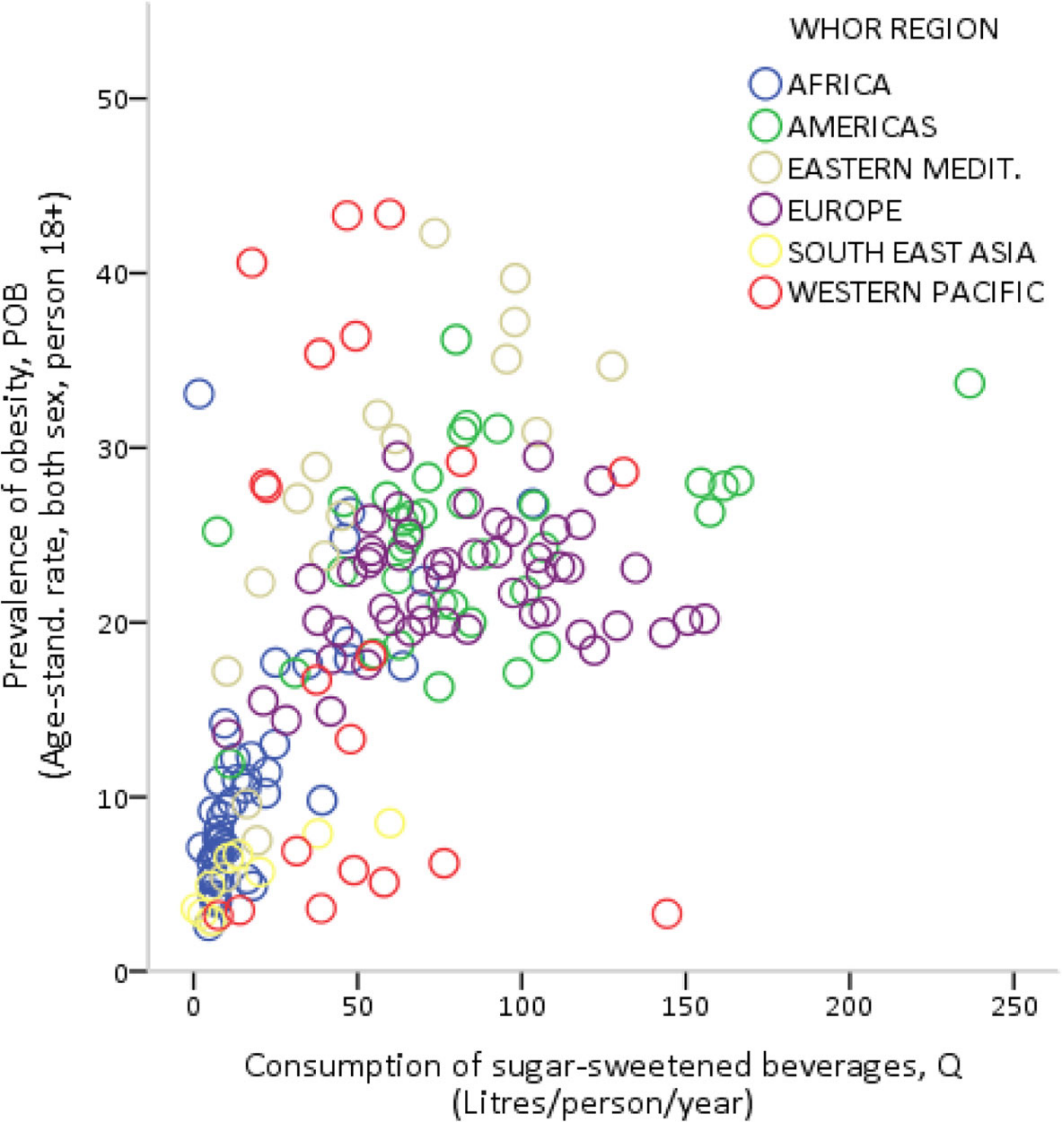
Sugar-sweetened beverage consumption and the prevalence of obesity

**Fig. 3:**
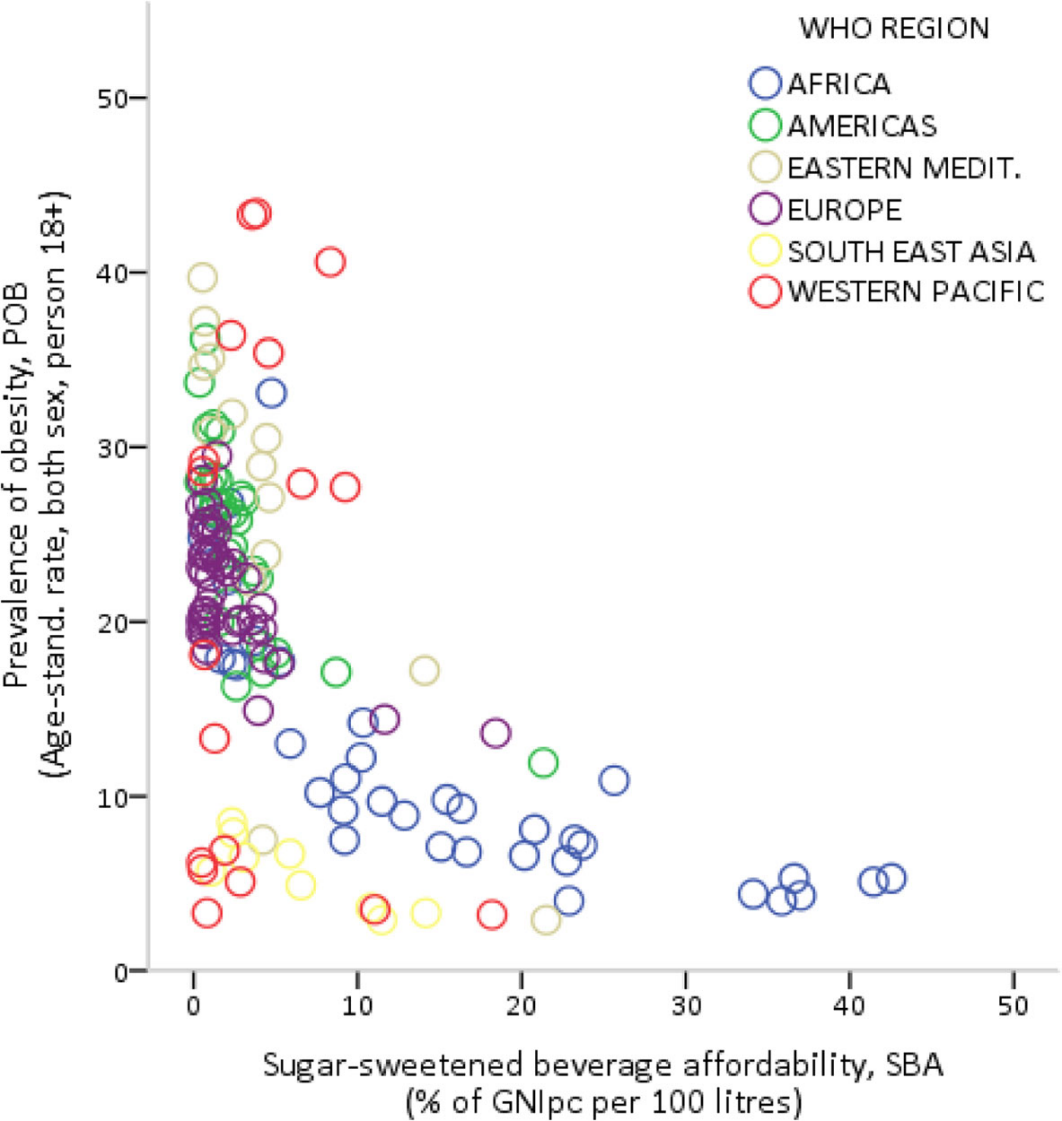
Sugar-sweetened beverage affordability and the prevalence of obesity

Figures 2 and 3 show the bivariate relationships between the age-standardized prevalence rate of obesity (*POB*) and, respectively, the consumption (*Q*) and affordability (*SBA*) of SSBs. In Fig. 2, the consumption of SSBs appears to be strongly and positively correlated with the prevalence of obesity (*r* = 0.64; *p* < 0.01, see Table 2), although the impact of consumption on obesity seems to increase at a decreasing rate. In Fig. 3, there is clear evidence that the prevalence of obesity decreases sharply as the relative income price of SSBs increases (*r* = 0.68; p < 0.01, see Table 2), especially in countries with values of *SBA* higher than 1 %. Similar results can be found using the prevalence rate of overweight instead of that of obesity (as shown in Figures S1 and S2 in Additional file 1).

To further explore these relationships, Tables 6 and 7 present the results of the multivariate regression models expressed by eqs. 3) and 4). Specifically, Table 6 refers to the effects of SSB consumption and affordability on overweight (eqs. 3*a* and 4*a*, respectively). In both equations, the goodness of fit was approximately 0.73, which suggests that the models could explain a relatively large part of the variation in the prevalence of overweight, and all coefficients—except for the quantity and price of bottled water—were statistically significant at 1 % (at 5 % only in the case of dietary energy supply in eq. 4*a*). The analysis supports the hypothesis of a harmful impact of SSB affordability on the spread of overweight. After controlling for the effects of various potential confounding factors, these results show that every 10 % increase in affordability (that is, every 10 % decrease in the relative income price of SSBs) was associated, on average, with about 0.4 more overweight adults per 100 inhabitants.

**Table 6:** Regression results: sugar-sweetened beverage consumption, affordability and overweight

Dependent variable: prevalence of overweight, POW				
Equation (3a)	Ln(·)	Coefficient	Std. Error^a^	t-statistic
Per capita consumption of sugar-sweetened beverages	Q	5.42**	1.31	4.13
Per capita consumption of water	QW	0.61	1.04	0.59
Dietary energy supply	DES	20.26**	7.06	2.87
Employment in services	ESE	13.92**	3.64	3.82
Gender inequality index	GII	5.53**	1.35	4.08
Number of physicians	PHY	2.20**	0.79	2.79
Constant = −188.42, F-statistic = 65.61 (p < 0.01), Adj. R-squared = 0.73, n = 144				
Equation (4a)	Ln(·)	Coefficient	Std. Error^a^	t-statistic
Sugar-sweetened beverage affordability	SBA	−4.09**	1.51	−2.72
(Average) Price of water	PW	0.29	2.03	0.14
Dietary energy supply	DES	16.21*	6.70	2.42
Employment in services	ESE	15.15**	4.52	3.35
Gender inequality index	GII	5.29**	1.33	3.97
Number of physicians	PHY	2.95**	0.83	3.53
Constant = −135.84, F-statistic = 54.72 (p < 0.01), Adj. R-squared = 0.73, *n* = 120				

Notes: ln(·) is natural log. * and ** denote *p* < 0.01 and *p* < 0.05, respectively

^a^White’s heteroskedasticity-adjusted standard errors

**Table 7:** Regression results: sugar-sweetened beverage consumption, affordability and obesity

Dependent variable: prevalence of obesity, POB				
Equation (3b)	Ln(·)	Coefficient	Std. Error^a^	t-statistic
Per capita consumption of sugar-sweetened beverages	Q	3.27**	0.77	4.27
Per capita consumption of water	QW	0.34	0.64	0.52
Dietary energy supply	DES	15.27**	4.11	3.71
Employment in services	ESE	8.91**	2.35	3.80
Gender inequality index	GII	4.68**	0.91	5.16
Number of physicians	PHY	0.61	0.49	1.24
Constant = 146.91, F-statistic = 48.35 (p < 0.01), Adj. R-squared = 0.67, *n* = 144				
Equation (4b)	Ln(·)	Coefficient	Std. Error^a^	t-statistic
Sugar-sweetened beverage affordability	SBA	−3.81**	1.07	−3.57
(Average) Price of water	PW	0.70	1.37	0.51
Dietary energy supply	DES	10.83**	4.00	2.71
Employment in services	ESE	8.93*	3.45	2.59
Gender inequality index	GII	5.21**	0.98	5.32
Number of physicians	PHY	0.60	0.54	1.11
Constant = −93.43, F-statistic = 38.70 (p < 0.01), Adj. R-squared = 0.66, n = 120				

Notes: ln(·) is natural log. * and ** denote p < 0.01 and p < 0.05, respectively

^a^White’s heteroskedasticity-adjusted standard errors

Table 7 refers to the effects of SSB consumption and affordability on the prevalence of obesity (eqs. 3*b* and 4*b*, respectively). Overall, the results are quite similar to those for overweight. There was a slight reduction in the coefficient of determination (the adjusted *R*^2^ is around 0.67), and the number of physicians was not statistically significant in both equations. However, all other regressors (except quantity and price of bottled water) were statistically significant and again displayed the expected sign. The magnitude of the impact of affordability on obesity was approximately the same as that on overweight (approximately 0.38 more cases of obesity per 100 adults for each 10 % increase in SSB affordability).

Finally, the regression analysis indicated that both the consumption and the price of bottled water were clearly unrelated to the prevalence of overweight and obesity. Although basic, this falsification test suggests that the observed harmful effects of SSB consumption and affordability on the population unhealthy weight outcomes were likely not due to some other omitted variables [28, 29].

## Discussion

The present study was designed to determine the effect of SSB affordability on the prevalence of overweight and obesity by using cross-sectional country data. The analysis revealed three main findings.

First, the affordability of SSBs showed both a high variability across countries and a clear tendency to increase substantially with the level of economic development. Second, affordability played a key role in determining cross-country differences in the quantity of SSBs consumed per capita. Third, there was a significant inverse relationship between the relative income price of SSBs and the prevalence of overweight and obesity; that is, the age-standardized prevalence rates of overweight and obesity increased with increasing affordability, all other things being equal.

A number of important limitations need to be acknowledged when interpreting these results. First, the total sugar content of the world’s most popular SSBs is on average approximately 10 g per 100 ml [53]. However, there are significant differences among the different types of products available—for instance between regular and diet sodas—that our analysis, based on aggregate market data, failed to take into account. Second, we computed consumption per capita starting from total sales in volume, which includes different levels of waste, and thus tends to overestimate the effective quantity consumed in each country. Third, our consumption and prevalence data are not fully comparable, because they relate to the total and adult populations, respectively. This mismatch might lead to a underestimation of the impact of SSBs on the population weight outcomes. Fourth, the adverse effects of SSBs on overweight and obesity are linked to their regular consumption over a long period of time, a phenomenon that should be better examined using panel data [29]. Finally, one might think of unhealthy eating habits and weight outcomes as being determined, at least partially, simultaneously, and this would suggest the use of a simultaneous equation model to capture reciprocal causality, as well as the inclusion of the price of other unhealthy foods correlated with SSB consumption. Unfortunately, the lack of reliable data for our very large set of countries makes such investigations infeasible.

**Fig. 4:**
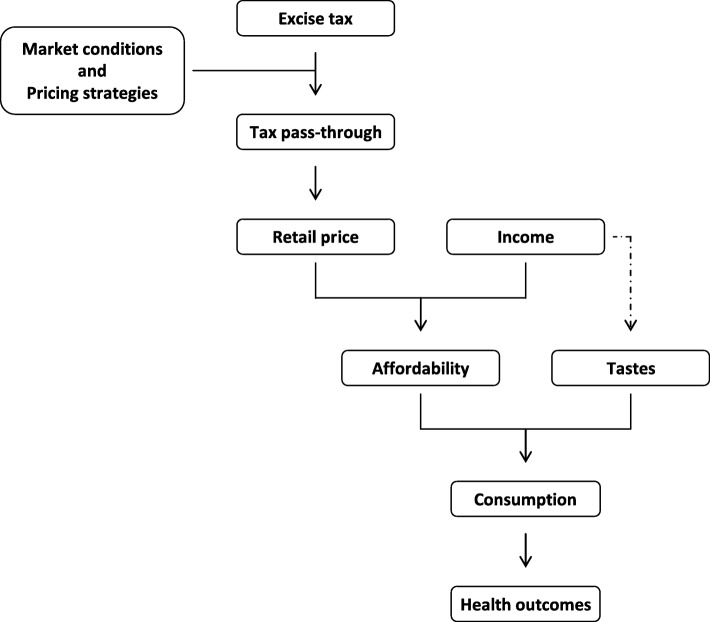
Sugar-sweetened beverage affordability and ‘soda taxes’

Despite these limitations, which are common to similar works [28], this study offers some useful insights for policymakers. In the context of the existing debate on how to curb the ‘obesity epidemic’ [54], our findings support current recommendations that fiscal policy should be included as part of a comprehensive strategy to prevent overweight and obesity [55]. This paper adds, as shown in Fig. 4, some empirical evidence that suggests the use of the affordability of SSBs, rather than just the price of SSBs, as an ‘intermediate target’ for health-related policies.

Figure 4 shows that the market conditions (i.e. price elasticities) and the firm’s pricing strategies determine how much of the excise tax is ‘passed through’ to consumers in a higher retail market price. Shelf price and the consumers’ disposable income, in turn, determine the level of affordability (i.e. the relative income price of SSBs). Finally, affordability and consumers’ tastes determine the purchasing behaviour and thus the quantity consumed.

The economic rationale behind the current choice to use excise taxes as a tool to control consumption lies in the price elasticity of the demand for these beverages that, despite some variability among studies, is frequently estimated [56] to be approximately 1 % or even higher (for instance, on average, 1.2% in the United States, France, Brazil, and Mexico [57]).

The fiscal approach, however, is based upon a static demand model, in which an increase in price, due to an excise tax, leads to a decrease in the quantity demanded; (that is, a movement along a given demand curve, where the consumers’ income and tastes remain unchanged). Instead, research has consistently shown that a key contributor to the obesity epidemic is that of structural changes in dietary patterns—i.e. the shift from traditional to Western-style diets—that result from the dynamic effects of rising income on consumers’ tastes and preferences in evolving technical and social environments [58].

Especially in growing economies, besides the question of how much of the tax burden falls on buyers as a higher retail price, relatively small changes in the nominal price of SSBs might be easily offset in the long-term by aggressive marketing strategies (e.g. indirect price discrimination through quantity discounts) and rising disposable income [59].

## Conclusions

By using Coca-Cola as a proxy for all SSBs, a recent study found that, during the last decades, SSBs have become more affordable around the world and especially in developing countries [30]. In this paper, we collected the latest statistics available on the beverage industry to compute SSB affordability for approximately 150 countries worldwide. These data were used to assess the impact of affordability—as measured by the SSB relative income price—on the prevalence of overweight and obesity. Our results clearly indicate that affordability: 1) is a major driver of purchasing behaviours, and 2) is significantly associated with the prevalence rates of both overweight and obesity.

This association obviously does not imply a causal relationship, and the use of country data offers the potential for ecological fallacies. However, soft drink affordability emerged as a reliable predictor of weight outcomes even after correcting for the main potential confounding factors. These results enhance our understanding of the harmful effects of SSBs and support the use of fiscal tools to control their consumption, by stressing the importance to focus on the effects of ‘soda taxes’ on the SSB relative income price^d.^^4^ However, there are still many intriguing issues which should be explored in further research, particularly to explain why in high-income countries very similar social and economic structures are associated with widely varying levels of SSB consumption.

## Additional file

Additional file 1: Including the following Tables and Figures: **Table S1.** Source and short description of each variable. **Table S2.** Raw data (full dataset, with 183 countries). **Figure S1.** Sugar-sweetened beverage consumption and the prevalence of overweight. **Figure S2.** Sugar-sweetened beverage affordability and the prevalence of overweight. (XLS 173 kb)

## Footnotes

1 The British Medical Association defines SSBs as all non-alcoholic water based beverages with added sugar, including sugar-sweetened soft drinks, energy drinks, fruit drinks, sports drinks and fruit juice concentrates [18].

2 Asian soft drinks are traditional or national (carbonated and non-carbonated) SSBs commonly found in several Western Pacific and Southern-East Asian countries (especially, in Japan, Malaysia, Indonesia, Thailand and the Philippines), such as ready-to-drink tea, cereal-pulse or milk based specialties, and many different type of local fruit-flavoured soda [20].

3 By using affordability in the demand function as an interactive term, we obtained the following results: 1) Q = 43.74–11.41P + 0.0009YPC - 9398.35(P/YPC) + 3.44PW + 63.49WLS (2.19) (0.0003) (2467.32) (3.10) (17.73) t = − 5.20 3.34–3.81 1.11 3.58 Adj. R^2^ = 0.68, *n* = 150 2) Q = 12.21–4.19P + 0.003YPC - 0.0005(P*YPC) + 1.65PW + 59.98WLS (2.62) (0.0006) (0.0001) (2.97) (17.45) t = − 1.60 4.61–3.38 0.56 3.44 Adj. R^2^ = 0.66, *n* = 150. Where White’s heteroskedasticity-adjusted standard errors are given parentheses. In each equation, the coefficient of the interactive term is significant at the 1% level. These results further support the use of the concept of affordability to investigate the consumption of SSBs.

4 It should be noted that, especially in high income countries, the major players in the beverage industry are currently trying to respond to public health concerns by replacing sugar (including high fructose corn syrup) with different types of artificial sweeteners [20]. From the perspective of public health, however, this marketing strategy could simply replace one problem with another, because of the potential harmful effects of these chemical additives on human health [60].
